# Unique local bone tissue characteristics in iliac crest bone biopsy from adolescent idiopathic scoliosis with severe spinal deformity

**DOI:** 10.1038/srep40265

**Published:** 2017-01-05

**Authors:** Zhiwei Wang, Huanxiong Chen, Y. Eric Yu, Jiajun Zhang, Ka-Yee Cheuk, Bobby K. W. Ng, Yong Qiu, X. Edward Guo, Jack C. Y. Cheng, Wayne Y. W. Lee

**Affiliations:** 1 SH Ho Scoliosis Research Laboratory, Department of Orthopaedics and Traumatology, Prince of Wales Hospital, The Chinese University of Hong Kong, Shatin, NT, Hong Kong SAR, China; 2 Joint Scoliosis Research Center of the Chinese University of Hong Kong and Nanjing University, The Chinese University of Hong Kong, Shatin, NT, Hong Kong SAR, China; 3 Bone Bioengineering Laboratory, Department of Biomedical Engineering, Columbia University, New York, USA; 4 Spine Surgery, Nanjing Drum Tower Hospital, Nanjing University, Nanjing, China

## Abstract

Adolescent idiopathic scoliosis is a complex disease with unclear etiopathogenesis. Systemic and persistent low bone mineral density is an independent prognostic factor for curve progression. The fundamental question of how bone quality is affected in AIS remains controversy because there is lack of site-matched control for detailed analysis on bone-related parameters. In this case-control study, trabecular bone biopsies from iliac crest were collected intra-operatively from 28 severe AIS patients and 10 matched controls with similar skeletal and sexual maturity, anthropometry and femoral neck BMD Z-score to control confounding effects. In addition to static histomorphometry, micro-computed tomography (μCT) and real time-PCR (qPCR) analyses, individual trabecula segmentation (ITS)-based analysis, finite element analysis (FEA), energy dispersive X-ray spectroscopy (EDX) were conducted to provide advanced analysis of structural, mechanical and mineralization features. μCT and histomorphometry showed consistently reduced trabecular number and connectivity. ITS revealed predominant change in trabecular rods, and EDX confirmed less mineralization. The structural and mineralization abnormality led to slight reduction in apparent modulus, which could be attributed to differential down-regulation of *Runx2*, and up-regulation of *Spp1* and *TRAP*. In conclusion, this is the first comprehensive study providing direct evidence of undefined unique pathological changes at different bone hierarchical levels in AIS.

Adolescent idiopathic scoliosis (AIS) is a three-dimensional (3D) spinal deformity occurring mainly in girls between ages 10 and 13 with a prevalence of 1–4% worldwide. Current treatment protocol is limited because of unclear etiopathogenesis. Various pathogenic mechanisms have been proposed^1^. Amongst, low bone mineral density (BMD), defined as Z-score of areal BMD (aBMD) at femoral neck by dual energy X-ray absorptiometry (DXA) ≤ −1 with reference to the age- and ethnic-matched population, has drawn specific attention because it was found to persist beyond skeletal maturity and identified as a significant prognostic factor for curve progression in 30–38% of AIS subjects^2^,^3^,^4^,^5^. With more advanced high resolution peripheral quantitative computed tomography (HR-pQCT) measurement, lower BMD and deranged trabecular bone microarchitecture was also found to be present in the distal radius of AIS subjects with normal BMD to less extent when compared with low BMD subgroup^6^. In addition, serological studies by us and others have reported higher serum level of bone specific alkaline phosphatase (bALP), nuclear factor kappa-B ligand (RANKL) and tartrate-resistant acid phosphatase 5b (TRAP5b) in AIS patient^7^,^8^,^9^. Furthermore, relative anterior spinal overgrowth determined by magnetic resonance imaging (MRI) was reported in AIS, suggesting asynchronous endochondral ossification^10^. These observed changes implied underlying abnormal systemic bone metabolism in AIS, which might result in decreased bone mechanical strength that could contribute to the initiation or progression of the spinal deformity^11^,^12^. To provide better understanding on the mechanism underlying these abnormal bone phenotypes in AIS which could be a novel therapeutic target, efforts have been spent to distinguish the specific bone-related features of AIS bone tissues, however, previous histomorphometry studies using human bone biopsies were with significant limitations due to either unmatched or lack of controls^13^,^14^.

In view of the lack of good animal model exhibiting anatomical feature of rotatory spinal deformity in AIS, human biopsies were primarily selected in this study. Despite vertebral column being the mostly affected bone site in AIS, it is practically and ethically difficult to collect bone biopsies from matched non-AIS adolescent for comparison. Given that trabecular bone in iliac crest share comparable compression strength and structural features to the spine^15^,^16^, it was chosen as alternative site for this study. It has been reported that rapid bone modeling and delayed mineral accrual during puberty might result in transient low bone mass and bone weakness^17^,^18^. Therefore, AIS and controls (Con) with similar aBMD were selected to control the confounding effect due to this significant bias. In addition to standard micro-computed tomography (μCT) analysis, finite element analysis (FEA), static histomorphometry and quantitative polymerase chain reaction (qPCR), an individual trabecula segmentation (ITS)-based morphological analysis and energy-dispersive X-ray spectroscopy (EDX) were used further to characterize individual trabecular type (plate and rod) and connectivity^19^, and relative calcium to phosphorous ratio. To the best of our knowledge, these techniques were used for the first time in AIS-related study to provide in-depth analysis on bone microstructure and mechanical properties, and bone metabolism related genes expression profile.

## Results

### Subjects characterization

The mean chronological age of AIS and Con subjects were 14.8 ± 1.89 and 14.5 ± 4.13 years old, respectively. There was no significant difference between AIS and Con in all the demographic and anthropometric parameters, and femoral neck BMD Z-score (Table 1).

### μCT and ITS analysis

Among all trabecular bone parameters evaluated from conventional μCT analysis (Table 2), AIS had slightly lower bone volume fraction (−5.26%, *p* = 0.405) and specific bone surface (−8.32%, *p* = 0.096), which was manifested as higher trabecular separation (18.75%, *p* = 0.033) and lower trabecular number (−17.03%, *p* = 0.064). The more obvious difference in connectivity density (Conn.D; −49.97%, *p* = 0.052) and structure model index (SMI; −13.17%, *p* = 0.711) between AIS and Con led to further analysis with ITS to reveal microstructural and connectivity changes in rod and plate trabeculae. ITS results (Table 3) revealed a predominate change in rod trabeculae bone volume fraction (−19.23%, *p* = 0.459), rod trabecular number (−11.04%, *p* = 0.229), and rod-rod junction density (−56.9%, *p* = 0.033) when compared with that of plate trabeculae.

### Finite element analysis

In view of the relations between trabecular bone microstructure and elastic modulus^20^,^21^, FE analysis was conducted to reveal if predominate changes in trabecular rods would affect the mechanical property of AIS bone. As shown in Table 4, the overall deranged trabecular plates and rods led to a trend of lower apparent modulus in AIS when compared with Con in both homogeneous and heterogeneous FE models, however, the difference did not reach statistical significance.

### Static bone histomorphometry

**Figure 1:**
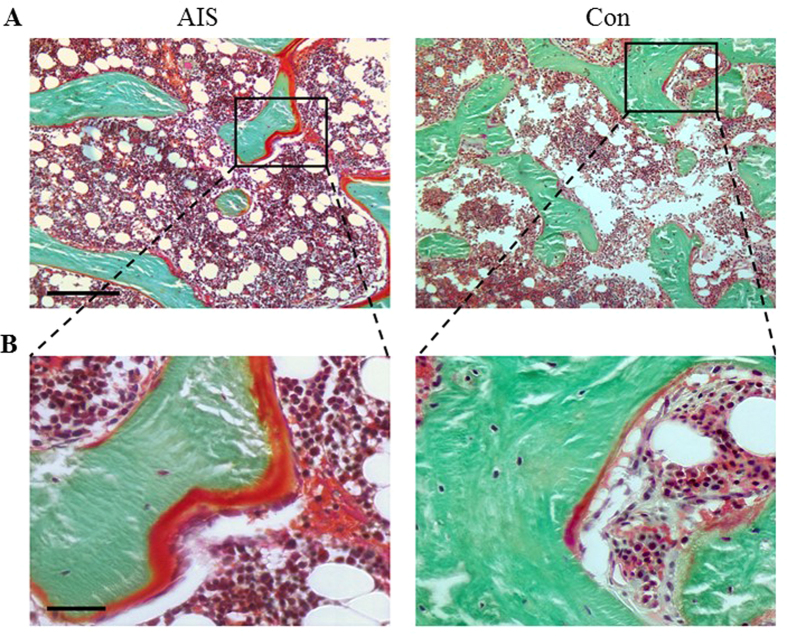
Representative Goldner’s Trichrome staining images of (**A**) AIS and (**B**) Con revealing higher osteoid volume, higher osteoblast number and lower osteocyte number in AIS. Scale bars, 250 μm and 50 μm (from top to bottom).

Similar to μCT analysis, structural indices of histomorphometric analyses (Fig. 1 and Table 5) revealed slightly lower bone volume fraction (−5.15%, *p* = 0.781) and trabecular number (−4.12%, *p* = 0.781), and higher trabecular separation (7.78%, *p* = 0.643). Cellular related parameters showed more osteoblasts number (105.8%, *p* = 0.033) and less osteocytes number (−21.65%, *p* = 0.116), but relatively similar osteoclasts number (−4.97%, *p* = 0.782). AIS had more osteoid volume (54.34%, *p* = 0.096) and osteoid width (25.68%, *p* = 0.926).

### Characterization of bone mineralization

As there is no normal reference reports for adolescents and young adults, we relied on previous reports on aged subjects to validate our findings. R_Ca/P_ value was reported to fall in the range of 1.65–1.9 in elderly with EDX measurement^22^,^23^ and was proportional to bone tissue age^24^. Our data of R_Ca/P_ (around 1.55) in AIS was in agreement with previous report of lower R_Ca/P_ value in younger bone tissue and indicated adequate homeostasis of calcium and phosphorous deposition in AIS. The ratio of calcium to phosphorus (R_Ca/P_) was widely accepted as an indicator for assessing the status of bone mineralization^25^,^26^. Carbon (C) was determined as the reference to assess the calcium content in the bone tissue because of its abundant and relatively stable content and being the major component of bone tissue^26^,^27^,^28^. EDX scanning showed significantly lower R_Ca/C_ in AIS, but there was absence of significant difference in R_Ca/P_ between AIS and Con, suggesting decreased mineralization which was in agreement with more osteoid as indicated by histomorphometric analyses (Table 6).

### mRNA levels of bone formation and resorption markers

At tissue level, AIS bone biopsies significantly expressed less *Runx2*, but more *Spp1* and *TRAP* when compared with Con. *ALP* and *CTSK* mRNA level in AIS were numerically lower in AIS, while *Col1* level was slightly increased in AIS numerically (Table 7).

## Discussion

AIS occurs in children during their pubertal growth spurt. Rapid growth is associated with the development and progression of scoliotic curves, with the curves stabilized at skeletal maturity^29^. Many previous studies had reported the association between AIS and low bone mineral density. Burner *et al*. was the first to report the relationship between osteoporosis and acquired back deformity in 1982^30^. Healey and Lane reported a higher prevalence of scoliosis in biopsy-proven osteoporotic women (48%)^31^. Cook *et al*. noted that AIS subjects had significantly lower lumbar spine and proximal femur BMD when compared with age-matched control subjects^32^. We investigated a large cohort of AIS and reported 36–38% of cases had generalized osteopenia (Z-score < −1)^3^. In a cross-sectional study on 919 girls with AIS, our group reported an inverse relationship between curve severity and BMD^33^. These observations have led researchers to investigate the roles of low bone mass and bone metabolism in the etiopathogenesis of AIS. More recent HR-pQCT studies revealed lower trabecular number^6^ and reduced computational determined apparent modulus in AIS^34^. It is speculated that such systemic abnormal bone quality and lower bone mineralization would make the AIS spine more vulnerable to deform once there is a certain degree of apical rotation of disproportionate or asymmetric skeletal growth^1^. However, the underlying mechanism remains poorly understood because of limited information on the pathogenic changes at bone tissue level. The present study, with age and gender, and bone site matched-controls and multiscale assessments at different bone hierarchical levels, revealed larger osteoid, deranged trabecular rods, abnormal osteoblasts and osteocytes number, and differentiated genes expression in AIS bone tissues, which could shed light on future mechanistic study.

ITS decomposing trabecular bone into trabecular rods and plates helped to pinpoint the predominant structural and connectivity changes of trabecular rods in AIS. Studies have shown that aging-related change towards more rod-like configuration could be associated with bone mechanical deterioration^35^,^36^. In contrary, the observed plate and rod configuration change in AIS was speculated to be different from other reports in premenopausal women with idiopathic osteoporosis^37^. Comparing with idiopathic osteoporosis with predominant reduction in pBV/TV (−41.5%) and pTb.N (−23.6%) but less in rBV/TV (−22.8%) and rTb.N (−10.2%), AIS showed relatively more reduction in rBV/TV (−19.2%), rTb.N (−11.04%) and rod to rod connectivity (−56.9%). Previous *in silico* study demonstrated that trabecular plate bone volume fraction was highly correlated to computationally determined anisotropic elastic moduli^19^. Further experimental study showed that trabecular plate-related parameters were highly correlated to elastic moduli and yield strength, while trabecular rod-related parameters were only slightly correlated to the mechanical properties^21^. In the present study, conventional homogenous FE analysis provided consistent result as previous study with HR-pQCT image obtained at distal radius^34^. More precise experimental setting with consideration of mineralization related tissue specific modules and heterogeneous FE analysis also resulted in similar finding.

Inadequate calcium and vitamin D intake/level are the major causes of osteomalacia in adult or rickets in children^38^,^39^. Although our data suggested osteomalacia-like phenotype in AIS, previous studies from us and other group did not show consistent difference in calcium or vitamin D intake between AIS and the controls. Lee *et al*. studied 596 AIS girls and 302 healthy control girls age 11–16 years old, and found that the mean calcium intake of both AIS and control groups reached only 36% and 32% of the Chinese calcium Dietary Reference Intake (DRI) of 1000 mg/day respectively^40^, and there was no different between the AIS and controls^4^. Yu *et al*. have reported slightly higher calcium intake in a case-control study of 214 AIS and 187 healthy girls aged 11 to 13 years old^41^. The median calcium intake was found to be 571.9 mg/day in the AIS group, and 587.9 mg/day in the control group, with no difference between the two groups. Despite the importance of vitamin D in bone mineralization, there is only one study reporting the dietary vitamin D intake in AIS patients and with small sample sizes. In addition to calcium intake, Akseer *et al*. have also studied the daily dietary vitamin D intake including supplement^42^. There was no difference in the dietary vitamin D intake between the groups of AIS patients with or without bracing and healthy controls. Interestingly, the lower R_Ca/C_ value suggests lower calcium content agreed with previous report of lower calcium deposition in AIS osteoblast culture^43^ and current data of altered bone metabolism related genes expression. Collectively, the higher osteoid and lower mineralization in AIS bone tissues are likely not following the same pathogenic pathway of osteomalacia although they shared some similar phenotypes.

The present study also for the first time reported significant up-regulation of bone formation (*Spp1*) and resorption (*TRAP*) genes, and down-regulation of *Runx2* mRNA in AIS bone biopsy. Runx2 is a key transcription factor regulating osteogenesis in response to canonical Wnt^44^ and transforming growth factor-beta (TGF-β)^45^, thus representing an early bone formation marker. The observed *Runx2* down-regulated expression in AIS suggests impaired osteogenic differentiation ability, which was in line with previous etiopathogenetic studies^46^,^47^. Osteoblasts regulate osteoclasts activities through the release of receptor activator of NF-κB ligand (RANKL) and osteoprotegerin (OPG). The RANKL/OPG ratio is an indicator of osteoclast differentiation and activation^48^. Our finding agreed with previous reported higher RANKL/OPG ratio in AIS girls^8^, pointing to the elevated bone resorption activity. It is speculated that the increased expression of bone resorption genes at tissue level might contribute to the reduction in rod trabecular number and rod related connectivity. It was speculated that the larger surface area to volume ratio of rod trabeculae could be more susceptible to bone resorption than the plate trabeculae, thus resulting in the observed structural changes above. It was suggested that the lower bone mass in AIS may result from sub-optimal bone mineralization qualitatively and quantitatively, and thus fails to catch-up with abnormally escalated bone growth during the peripubertal period. Our on-going study on osteocytes suggests impaired differentiation from osteoblasts to osteocytes via aberrant miRNA signaling might partly explain the lower mineralization and higher osteoid in the present study.

To the best of our knowledge, this is the first comprehensive *ex vivo* study providing direct evidence of previously undefined unique bone microstructure, mechanical property, mineralization and local gene expression profile in AIS with matched-controls at bone tissue level, which could shed light on future mechanistic and therapeutic studies via targeting on the bone metabolism and mineralization pathways. However, the present study was limited by the small sample size though comparable to similar studies in adolescents^49^,^50^. Moreover, as tetracycline administration for dynamic histomorphometry analysis has been ethically prohibited, it would not be possible to directly look into the rates of mineral apposition, bone formation and resorption quantitatively. Recent advancement has enabled non-invasive time-lapse monitoring of bone formation and resorption in human subjects^51^, which would provide novel insight into the link between abnormal bone metabolism and low BMD in AIS during curve progression. In addition, it is of great clinical importance if improving bone quality could be an effective intervention to reduce the risk of curve progressing to the surgical threshold, i.e. beyond 45° Cobb angle^52^. Previous proof-of-concept study with whole body vibration showed that aBMD in AIS girls (15 to 25 years old) could be improved, which however is ethically not applicable for younger subjects with mild curve because of potential undesired effect on growth plates and skeletal growth^53^. Our ongoing randomized controlled study with calcium and vitamin D supplementation might help to fill up this considerable gap of knowledge of clinical impact (unpublished work).

## Methods

### Subjects

From July of 2012 to August of 2014, twenty eight female AIS patients^54^ with severe scoliosis undergoing posterior instrumentation and spinal fusion surgery were recruited from our center. The diagnosis was confirmed clinically and radiologically with standing full-spine posteroanterior (PA) X-ray radiograph after excluding other possible causes of scoliosis.

Due to the difficulty and ethical problems in collecting fresh bone biopsy from non-AIS adolescent, the number of controls recruited was in about 1:3 ratio. During the reported period, ten age-, gender- and ethnic-matched adolescents (Con) without diseases affecting bone metabolism and in need of iliac crest autograft as part of their orthopaedic surgical procedure were recruited as controls. The exclusion criteria were as follows: (a) other scoliosis, such as congenital scoliosis, neurofibromatosis, ankylosing spondylitis, syringomyelia with scoliosis; (b) any medical conditions that affected bone metabolism, such as hyperparathyroidism and acute or chronic renal disease; (c) receiving drug treatment that affected bone metabolism such as bisphosphonate and steroid; (d) history of operation that would affect bone metabolism such as the hepatic surgery; (e) malignant bone tumors such as osteosarcomas; (f) presence of any implants that affected BMD measurement; (g) pregnancy or malabsorption history.

Clinical ethical approval in comply with the Declaration of Helsinki was obtained from the Ethical Committee of the university and hospital (Reference number: CREC-2012.528). Details of the research project and biopsy collection were explained to all subjects and/or their parents or guardians before entering into the study. Informed consent was obtained from all subjects. Standardised trabecular bone biopsies of less than 5 × 5 × 5 mm^3^ were obtained from the posterior part of the ilium of AIS patients and controls at 2 cm below iliac crest and 2 cm in front of the posterior-superior iliac spine intraoperatively as part of the autograft harvesting procedure. Due to the small tissue size, the patients were randomly divided into two groups: a) bone tissues subjected to μCT scanning (including ITS and FE analysis) and then embedded for static bone histomorphometry and EDX scanning; and b) bone tissues for total RNA extraction.

### Demographic, anthropometric and radiological assessments

Anthropometric parameters including body weight, standing height and arm span were measured with standard stadiometry techniques^55^. Arm span was used for the calculation of body mass index (BMI = bodyweight/armspan^2^) to minimize the inaccuracy in the formula based estimation of the corrected standing height in AIS^56^. Tanner stage and Risser grade were used for the assessment of the sexual and bone maturity. Curve severity was graded with standard Cobb angle of the major curve on the standard full-spine radiograph.

### Areal BMD (aBMD) measurements

Due to the axial vertebral rotation in transverse plan, measurement of bone mineral content per unit projectional area in AIS spine would be significantly biased^57^. Therefore, aBMD (g/cm^2^) of the femoral neck at the concave side of the major curve in AIS was measured by DXA (XR-36; Norland Medical Systems, Fort Atkinson, WI, USA). For controls, aBMD was measured at the non-dominant femoral neck. A normative BMD dataset of local ethnic Chinese was used for the calculation of the age- and gender-adjusted femoral neck BMD Z score^5^.

### Micro-CT measurements

A standard measurement protocol was adopted for the scanning of the entire biopsy with source energy at a voltage of 70 kVp with a current of 114 μA (μCT 40, Scano, Brüttisellen, Switzerland). The entire biopsies were scanned with the voxel size at 10 μm^58^. As to standardize the volume of interest in evaluation, a 4 × 4 × 4 mm^3^ cubic sub-volume of samples was contoured for subsequent μCT standard evaluation, ITS analysis and FEA. Low-pass Gaussian filter (Sigma = 0.8; Support = 1) was used to remove the noise signal. A sample specific adaptive thresholding technique was applied to binarize gray scale image using the standard protocol of Scanco μCT analysis. BV/TV (bone volume fraction), Tb.N (trabecular number, mm^−1^), Tb.Th (trabecular thickness, mm), Tb.Sp (trabecular separation, mm), SMI (structure model index), Conn.D (connectivity density, mm^−3^), BS/BV (specific bone surface, mm^2^/mm^3^), vBMD (average volumetric bone mineral density, mgHA/cm^3^) and mBMD (material bone tissue mineral density, mgHA/cm^3^) were acquired for analysis.

### ITS-based morphological analysis of micro-CT

ITS analysis was performed as previously described^19^. Briefly, the 3D segmented trabecular bone images were completely decomposed by digital topologic analysis (DTA)-based skeletonization and reconstructed by iterative reconstruction method with a validated software developed from Bone Bioengineering Laboratory of Columbia University. Based on the evaluations of dimension and orientation of each individual trabecular plate and rod, as well as junctions of surface and curve skeletons, a set of ITS-based morphological parameters was derived to quantify total, plate and rod bone volume fraction (BV/TV, pBV/TV and rBV/TV), plate to rod ratio (P-R ratio), that is, the ratio of plate bone tissue divided by rod bone tissue, plate and rod numerical density (pTb.N and rTb.N, mm^−1^), plate and rod thickness (pTb.Th and rTb.Th, mm), plate surface area (pTb.S, mm^2^), rod length (rTb.L, mm), and rod-rod, plate-rod, and plate-plate junction density (R-R, P-R, and P-P Junc.D, mm^−3^).

### Finite element analysis-based determination of bone mechanical properties

Three finite element (FE) models were generated for each specimen with different bone tissue properties: (1) homogeneous model with a global constant and routinely used tissue modulus; (2) homogeneous model with a sample specific tissue modulus scaled by the average tissue mineral density (TMD); and (3) a heterogeneous model. The cubic sub-volume of μCT images were used to perform linear FEA to determine the trabecular bone elastic mechanical property. In the specimen-specific homogeneous FE, different tissue modulus was assigned to each specimen with the mean Young’s modulus in the heterogeneous FE model^59^. In the heterogeneous FE model, the Young’s modulus of each bone element was assigned into different value by using a linear attenuation-to-tissue modulus relationship^59^. The bone in both FE models was defined as an isotopic material with a Poisson’s ratio of 0.3^60^. An uni-axial compression test was simulated to calculate apparent modulus as expression of overall trabecular bone elastic mechanical property.

### MMA embedding and histomorphometry

The bone tissues were fixed in 70% ethanol overnight and then embedded in Technovit 9100 (methylmethacrylate, MMA, HeraeusKulzer, Wehrheim, Germany) according to manufacturer’s instruction. The embedded bone tissues were trimmed with diamond band cutting device (EXAKT 300 CP, EXAKT Apparatebau GmbH, Horderstedt, Germany) to obtain cylindroid samples. the embedded tissues were cut into 5 um think sections with rotary microtome (RM2255, Leica) with carbide knives (Slee, Mainz, Germany), and then mounted on coated glass slide. 5 um thick bone slices were stained with Goldner’s trichrome method. For each sample, 20 images of 200X The areas of mineralized and non-mineralized region stained in green and red, and other static histomorphomteric parameters were evaluated as previously stated with osteospecific image analysis software (Osteomeasure, OsteoMetrics Inc, GA, USA)^61^.

### Calcium and phosphorus content measurement with EDX

MMA-embedded bone biopsy was sectioned at 100 μm thickness with circular saw (SP1600, Leica). Sectioned biopsy surfaces were grinded with sand paper with increasing grid number, and then coated with a thin gold layer by vacuum evaporation Edwards sputter coater (Electron Microscopy Sciences, Hatfield, PA, USA)^62^. Calcium and phosphorous contents were measured with digital SEM (SU8010, Hitachi, Japan) equipped with EDX (IXRF Systems, Austin, TX, USA). The accelerating voltage was set at 10 kV, probe current at 10 μA, working distance at 15 mm and pixel resolution at 1.0–1.3 nm/pixel. For each specimen, five trabecular bone fragments were selected, and three ROIs (0.1 × 0.1 mm^2^) was randomly selected on each fragment. 5 out of 14 AIS bone sections were not large enough to fulfil ROI selection criteria. The microprobe was calibrated with a take-off angle of 35° and an acquisition time of 100 s. The relative weight of calcium (rWt%Ca) and phosphorus (rWt%P) was acquired with built-in software and the value of calcium to phosphorus ratio (R_Ca/P_) was determined. The relative weight of carbon (rWt%C) was used as background control for the calculation of calcium to carbon ratio (R_Ca/C_).

### Total RNA extraction and real time qPCR

Bone tissue was grounded with autoclaved pestle and mortar. Total RNA was extracted with Trizol (Life Technologies, Carlsbad, California, USA) according to manufacturer’s protocol. RNA quality was evaluated with NanoDrop^®^ ND-2000 (Thermo Scientific, Waltham, Massachusetts, USA). Only RNA with 260/280 value equal or over 1.8 was used in subsequent experiments. 200 ng RNA was reversed transcripted into cDNA by Takara reverse transcription kit (Takara-bio, Shiga, Japan). Real time qPCR was performed using Power SYBR^®^ Green (Life Technologies, Carlsbad, California, USA) according to manufacturer’s instruction. The qPCR reaction was performed with the following condition: 94 °C for 10 min followed by 40 cycles of 95 °C for 15 s (denaturation) and 60 °C for 30 s (annealing and elongation). RNase-free water was used as negative control. The relative expression level of the genes normalized to glyceraldehyde 3-phosphate dehydrogenase (GAPDH) was calculated with the 2^−ΔΔCT^ formula. The primers sequences and related information were listed in Table 8.

### Statistical analysis

All data is expressed as mean ± SD. Mann–Whitney *U* test was used for testing. All the statistical analysis was performed with SPSS 17.0 software (SPSS, Inc., Chicago, IL, USA). *P* < 0.05 was considered statistically significant.

## Additional Information

**How to cite this article**: Wang, Z. *et al*. Unique local bone tissue characteristics in iliac crest bone biopsy from adolescent idiopathic scoliosis with severe spinal deformity. *Sci. Rep.* **7**, 40265; doi: 10.1038/srep40265 (2017).

**Publisher's note:** Springer Nature remains neutral with regard to jurisdictional claims in published maps and institutional affiliations.

## Figures and Tables

**Table 1:** Demographic, anthropometric and femoral neck BMD Z-score in AIS and controls.

Parameters	AIS N = 28	Con N = 10	*p* value
Age (years)	14.8 ± 1.89	14.5 ± 4.13	0.591
Major curve (°)	52.7 ± 10.05	—	—
Risser grade	3.4 ± 1.31	3.4 ± 1.43	0.827
Tanner stage	3.0 ± 1.10	2.6 ± 1.67	0.497
Standing height (cm)	161.5 ± 8.04	161.2 ± 13.66	0.864
Body weight (kg)	47.5 ± 9.57	51.3 ± 14.37	0.483
Arm span (cm)	160.5 ± 9.04	159.9 ± 10.2	0.891
BMI (kg/m^2^)	18.5 ± 4.23	20.0 ± 5.24	0.452
Femoral neck BMD Z-score^#^	−0.96 ± 0.72	−0.91 ± 1.19	0.751

Note: Difference % = (AIS − control)/(control) %.

**Table 2:** Bone trabecular micro-architecture measured with μCT in AIS and controls.

Parameters	AIS *N* = 14	Con *N* = 5	% difference	*p* value
BV/TV(%)	0.18 ± 0.033	0.19 ± 0.035	−5.26	0.405
Tb.N (mm^−1^)	1.90 ± 0.271	2.29 ± 0.422	−17.03	0.064
Tb.Th (mm)	0.12 ± 0.016	0.114 ± 0.011	5.26	0.308
Tb.Sp (mm)	0.57 ± 0.094	0.48 ± 0.085	18.75	0.033
SMI	0.79 ± 0.319	0.91 ± 0.176	−13.17	0.711
BS/BV (mm^2^/mm^3^)	21.28 ± 2.431	23.21 ± 1.179	−8.32	0.096
Conn.D (mm^−3^)	15.95 ± 4.993	31.88 ± 25.761	−49.97	0.052
vBMD (mgHA/cm^3^)	137.88 ± 30.152	152.23 ± 37.860	−9.43	0.517
mBMD (mgHA/cm^3^)	634.98 ± 33.559	629.52 ± 40.824	0.87	0.926

Note: BV/TV is bone volume fraction; Tb.N is trabecular number; Tb.Th is trabecular thickness; Tb.Sp is trabecular separation; SMI is structure model index; Conn.D is connectivity density; BS/BV is specific bone surface; vBMD is average volumetric bone mineral density and mBMD is material bone tissue mineral density. Difference % = (AIS − control)/(control) %.

**Table 3:** Trabecular micro-architecture measured with ITS in AIS and controls.

Parameters	AIS *N* = 14	Con *N* = 5	% difference	*p* value
pBV/TV	0.16 ± 0.027	0.17 ± 0.026	−5.88	0.517
rBV/TV	0.021 ± 0.008	0.026 ± 0.014	−19.23	0.459
P-R ratio	7.93 ± 1.928	7.31 ± 2.305	8.48	0.781
pTb.N (mm^−1^)	4.10 ± 0.27	4.38 ± 0.387	−6.39	0.139
rTb.N (mm^−1^)	2.90 ± 0.317	3.26 ± 0.716	−11.04	0.229
pTb.Th (mm)	0.087 ± 0.003	0.082 ± 0.003	6.1	0.012
rTb.Th (mm)	0.065 ± 0.003	0.062 ± 0.004	4.84	0.052
pTb.S (mm^2^)	0.026 ± 0.002	0.024 ± 0.003	8.33	0.165
rTb.L (mm)^#^	0.215 ± 0.012	0.211 ± 0.004	1.9	0.926
R-R Junc.D (mm^−3^)^#^	7.84 ± 2.568	18.19 ± 18.765	−56.9	0.033
R-P Junc.D (mm^−3^)	76.82 ± 24.618	103.82 ± 56.032	−26	0.267
P-P Junc.D (mm^−3^)	71.34 ± 18.528	94.57 ± 40.344	−24.56	0.229

Note: pBV/TV is plate bone volume fraction; rBV/TV is rod bone volume fraction; P-R ratio is the ratio of plate bone tissue to rod bone tissue; pTb.N is plate trabeculae number density; rTb.N is rod trabeculae number density; pTb.Th is mean trabecular plate thickness; rTb.Th is mean trabecular rod thickness; pTb.S is mean trabecular plate surface area; rTb.L is mean trabecular rod length; R-R Junc.D is rod-rod junction density; P-R Junc.D is plate-rod junction density and P-P Junc.D is plate-plate junction density. Difference % = (AIS − control)/(control) %.

**Table 4:** Apparent modulus in AIS and controls calculated by homogeneous and heterogeneous finite element analysis (FEA).

Parameters	AIS *N* = 14	Con *N* = 5	% difference	*p* value
Apparent modulus (MPa) by homogeneous FEA experimentally obtained average modulus as Young’s modulus	96.78 ± 47.04	107.20 ± 36.67	−9.72	0.517
Apparent modulus (MPa) by homogeneous FEA Sample specific modulus	97.41 ± 49.62	105.57 ± 33.11	−7.73	0.355

Note: Difference % = (AIS − control)/(control) %.

**Table 5:** Bone histomorphometric parameters determined with Osteomeasure in AIS and controls.

Parameters	AIS *N* = 14	Con *N* = 5	% difference	*p* value
BV/TV [%]	26.54 ± 6.425	27.98 ± 7.004	−5.15	0.781
Tb.N [/mm]	1.94 ± 0.450	2.03 ± 0.403	−4.12	0.781
Tb.Wi [um]	137.62 ± 20.590	138.59 ± 24.566	−0.70	0.781
Tb.Sp [um]	401.90 ± 116.253	372.90 ± 116.861	7.78	0.643
O.Wi [um]	16.53 ± 16.458	13.16 ± 5.168	25.68	0.926
OS/BS [%]	15.98 ± 7.272	10.56 ± 7.702	51.31	0.165
OV/BV [%]	2.60 ± 1.168	1.69 ± 1.231	54.34	0.096
Ob.S/BS [%]	9.30 ± 4.489	5.72 ± 0.874	62.77	0.166
N.Ob/BS [/mm]	8.93 ± 3.220	4.34 ± 1.013	105.80	0.033
Oc.S/BS [%]	1.14 ± 0.456	1.21 ± 0.276	−5.71	0.926
N.Oc/BS [/mm]	0.328 ± 0.121	0.345 ± 0.153	−4.97	0.782
N.Ot/BAr [/mm2]	166.92 ± 38.103	213.05 ± 64.889	−21.65	0.116

Note: BV/TV is bone volume fraction; Tb.N is trabecular number; Tb.Wi is trabecular width; Tb.Sp is trabecular separation; O.Wi is osteoid width; OS/BS is osteoid surface; OV/BV is osteoid volume; Ob.S/BS is osteoblast surface; N.Ob/BS is osteoblast number; Oc.S/BS is osteoclast surface; N.Oc/BS is osteoclast number; N.Ot/Bar is number of osteocytes per bone area. Difference % = (AIS − control)/(control) %.

**Table 6:** Bone mineral status measured with SEM/EDX in AIS and controls.

Parameters	AIS *N* = 9	Con *N* = 5	*p* value
R_Ca/P_	1.55 ± 0.0542	1.56 ± 0.041	0.947
R_Ca/C_	0.77 ± 0.13	1.05 ± 0.1	0.006

**Table 7:** Local expression level of bone formation and resorption related mRNA in AIS and controls.

mRNA (relative to *GAPDH)*	AIS *N* = 14	Con *N* = 5	% difference	*p* value
*Runx2*	0.016 ± 0.0075	0.027 ± 0.0078	−40.74	0.012
*ALP*	0.017 ± 0.0209	0.024 ± 0.0108	−29.17	0.179
*Col1*	0.502 ± 0.5269	0.255 ± 0.4195	96.86	0.106
*Bglap*	0.024 ± 0.0357	0.007 ± 0.0051	242.86	0.244
*Spp1*	0.354 ± 0.3455	0.085 ± 0.1618	316.47	0.022
*TRAP*	0.016 ± 0.0109	0.006 ± 0.0116	166.67	0.041
*CTSK*	0.027 ± 0.0269	0.010 ± 0.0160	170	0.101

Note: Difference % = (AIS − control)/(control) %.

**Table 8:** Primers sequences for qPCR.

Name	Protein name	Sequences: Forward (F) and Reverse (R)	Size (bp)	NCBI number
*Runx2*	Runt-related transcription factor 2	F: 5′-GACAAGCACAAGTAAATCATTGAACTACAG-3′ R: 5′-GTAAGGCTGGTTGGTTAAGAATCTCTG-3′	196	NM_001024630.3
*ALP*	Alkaline phosphatase	F: 5′-GGACACTGGGCATAGATTTCTCAAG-3′ R: 5′-TGCTGGGATTACAAACACTTCCTTT-3′	109	NM_001632.4
*Col1*	Collagen type I	F: 5′-GTCACCCACCGACCAAGAAACC-3′ R: 5′-AAGTCCAGGCTGTCCAGGGATG-3′	121	NM_000088.3
*Bglap*	Osteocalcin	F: 5′-CCTCACACTCCTCGCCCTATT-3′ R: 5′-CCCTCCTGCTTGGACACAAA-3	117	NM_199173.5
*Spp1*	Osteopontin	F: 5′-GTACCCTGATGCTACAGACG-3′ R: 5′-TTCATAACTGTCCTTCCCAC-3′	139	NM_001040058.1
*TRAP*	Tartrate-resistant acid phosphatase	F: 5′-TGACAAGAGGTTCCAGGAGAC-3′ R: 5′-GAAGTGCAGGCGGTAGAAAG-3′	178	NM_001111035.1
*CTSK*	Cathepsin K	F: 5′-AACGAAGCCAGACAACAGATT-3′ R: 5′-CGAGAGATTTCATCCACCTTGTT-3′	170	NM_000396.3
*GAPDH*	Glyceraldehyde 3-phosphate dehydrogenase	F: 5′-TGCACCACCAACTGCTTAGC-3′ R: 5′-GGCATGGACTGTGGTCATGAG-3′	178	NM_002046.5
